# Characteristics and Factors for Short-Term Functional Outcome in Stroke Patients With Atrial Fibrillation, Nationwide Retrospective Cohort Study

**DOI:** 10.3389/fneur.2019.01101

**Published:** 2019-10-18

**Authors:** Tae-Jin Song, In-Young Baek, Ho Geol Woo, Yong-Jae Kim, Younkyung Chang, Bum Joon Kim, Sung Hyuk Heo, Jin-Man Jung, Kyungmi Oh, Chi Kyung Kim, Sungwook Yu, Kwang Yeol Park, Jeong-Min Kim, Jong-Ho Park, Jay Chol Choi, Man-Seok Park, Joon-Tae Kim, Kang-Ho Choi, Yang-Ha Hwang, Jong-Won Chung, Oh Young Bang, Gyeong-Moon Kim, Woo-Keun Seo

**Affiliations:** ^1^Department of Neurology, College of Medicine, Ewha Womans University Mokdong Hospital, Seoul, South Korea; ^2^Department of Neurology, Samsung Medical Center, Sungkyunkwan University School of Medicine, Seoul, South Korea; ^3^Department of Digital Health, SAIHST, Sungkyunkwan University, Seoul, South Korea; ^4^Department of Neurology, Eunpyeong St.Mary's Hospital, The Catholic University of Korea, Seoul, South Korea; ^5^Department of Neurology, Kyung Hee University College of Medicine, Seoul, South Korea; ^6^Department of Neurology, Korea University Ansan Hospital, Korea University College of Medicine, Seoul, South Korea; ^7^Department of Neurology, Korea University Guro Hospital, Korea University College of Medicine, Seoul, South Korea; ^8^Department of Neurology, Korea University Anam Hospital, Korea University College of Medicine, Seoul, South Korea; ^9^Department of Neurology, Chung-Ang University College of Medicine, Seoul, South Korea; ^10^Department of Neurology, MyongjiHospital, Hanyang University, College of Medicine, Goyang-si, South Korea; ^11^Department of Neurology, Jeju National University, Jeju-si, South Korea; ^12^Department of Neurology, Chonnam National University Medical School and Hospital, Gwangju, South Korea; ^13^Department of Neurology, School of Medicine, Kyungpook National University, Daegu, South Korea

**Keywords:** atrial fibrillation, stroke, Korea, nationwide cohort, outcome assessment

## Abstract

**Background and aims:** Atrial fibrillation (AF) is a major cause of ischemic stroke; however, detailed clinical data and prognostic factors for stroke patients with AF are lacking in Korea. We aimed to investigate clinical information and factors associated with functional outcomes of stroke patients with AF from the Korean nationwide ATrial fibrillaTion EvaluatioN regisTry in Ischemic strOke patieNts (K-ATTENTION) database.

**Methods:** From January 2013 to December 2015, consecutive clinical information from acute stroke patients with AF or history of AF was collected from 11 centers in Korea. Collected data included demographics, risk factors, pre-stroke medication, stroke severity, stroke subtypes, concomitant cerebral atherosclerosis, brain image findings, recanalization therapy, discharge medication, and functional outcome at 3 months after index stroke.

**Results:** A total of 3,213 stroke patients (mean age, 73.6 ± 9.8 years; female, 48.6%) were included. The mean CHA_2_DS_2_-VASc score was 4.9. Among the 1,849 (57.5%) patients who had brain image and functional outcome data, poor outcome (modified Rankin scale > 2) was noted in 53.1% (981/1,849) of patients. After adjusting for age, sex, and variables that had a *p* < 0.05 in univariate analysis or well-known factors for functional outcome, presence of asymptomatic extracranial cerebral atherosclerosis [odd ratio (OR): 1.96, 95% confidence interval (CI): 1.36–2.82, *p* = 0.001] and less frequent prior stroke statin intake (OR: 0.69, 95% CI: 0.49–0.98, *p* = 0.038) were associated with poor functional outcome.

**Conclusion:** Our results suggest that presence of non-relevant extracranial cerebral atherosclerosis may affect poor functional outcome and prior stroke statin therapy may be feasible in Korean stroke patients with AF.

## Introduction

Atrial fibrillation (AF) is frequent cardiac arrhythmia and is associated with increased risk of systemic embolism, hospitalization, and death (1, 2). Particularly, AF is a well-known major risk factor for occurrence and recurrence of stroke and stroke-related mortality (3). With the current aging population and associated increased concomitant vascular risk factors and/or cardiovascular disease, the burden of AF is increasing in the Western and Asian populations, including South Korea, China, and Japan (4–7).

Meanwhile, most of the current data on AF patients with a previous history of stroke have focused on secondary prevention from recurrent ischemic stroke or other thromboembolic complications; in contrast, evidence regarding acute stroke patients with AF has been limited. The functional outcome of AF patients with acute stroke is a major determinant of mortality and long-term recurrence and the decision whether to use oral anticoagulants is dependent on the neurological status and findings obtained from neuroimaging rather than conventional risk stratification schemes such as CHA_2_DS_2_-VASc score. Therefore, comprehensive real-world data considering neurological status, acute treatment pattern such as reperfusion therapy, neuroimaging findings, and early outcome of acute stroke in patients with AF is needed. Furthermore, although characteristics or outcome of stroke may be different according to race in stroke patients with AF, researches regarding characteristics and factors for short-term functional outcome of AF-related stroke for in Asian stroke population have been limited.

We aimed to demonstrate demographics and outcome parameters and to investigate factors associated with early functional outcomes of stroke patients with AF from the Korean nationwide ATrial fibrillaTion EvaluatioN regisTry in Ischemic strOke patieNts (K-ATTENTION) database.

## Materials and Methods

### Study Design

The K-ATTENTION is a multicenter, nationwide, retrospective cohort study to evaluate the diagnosis, treatment, and prognosis of acute stroke subjects with AF in a real-world clinical setting in Korea from January 2013 to December 2015. Regional and local emergency medical centers (9 tertiary and 2 secondary hospitals) from 5 provinces participated in this registry. The list of participating hospitals and numbers of included subjects are shown in Supplementary Figure 1. Criteria for inclusion are presented in Supplementary Table 1. Approximately 80% of stroke subjects who were admitted to each hospital were from the city, county, or district where the patient lives. In our registry, no subjects were registered in duplicate. The comparison between K-ATTENTION and other previous researches regarding stroke subjects with AF is shown in Supplementary Table 2. The primary, secondary, and exploratory purpose of our study and plans of our research are described in Supplementary Table 3. The design of this study was approved by the Institutional Review Board from each participating site. Because of the retrospective and observational nature of the protocol, written informed consents were waived (Samsung Medical Center, 2016-07-011).

### Subjects

As described in Supplementary Table 1, all of subjects over 20 years of age who were diagnosed with cerebral infarction accompanying AF during the study period (January 1, 2013 to December 31, 2015) or having past history of AF were consecutively included in the study from each participating center. Cerebral infarction was defined as ischemic lesions as identified through brain imaging (CT or MRI) accompanied by neurologic symptoms or signs. The presence of AF was defined as arrhythmia that lasted for more than 30 s with irregular R-R intervals, no clear repetitive P-waves, and cardiologist confirmation at each hospital. AF was diagnosed via electrocardiogram (ECG), 24 or 48-h electrocardiogram, or telemonitoring with recording and automated rhythm detection. Subjects who were not adequately screened for stroke and arrhythmia (for example, subjects that did not undergo brain CT and/or MRI, MRA, or ECG) or without evidence of cerebral infarction on brain images were excluded from the study (8–10).

### Clinical Data and Outcome Collection

**Figure 1:**
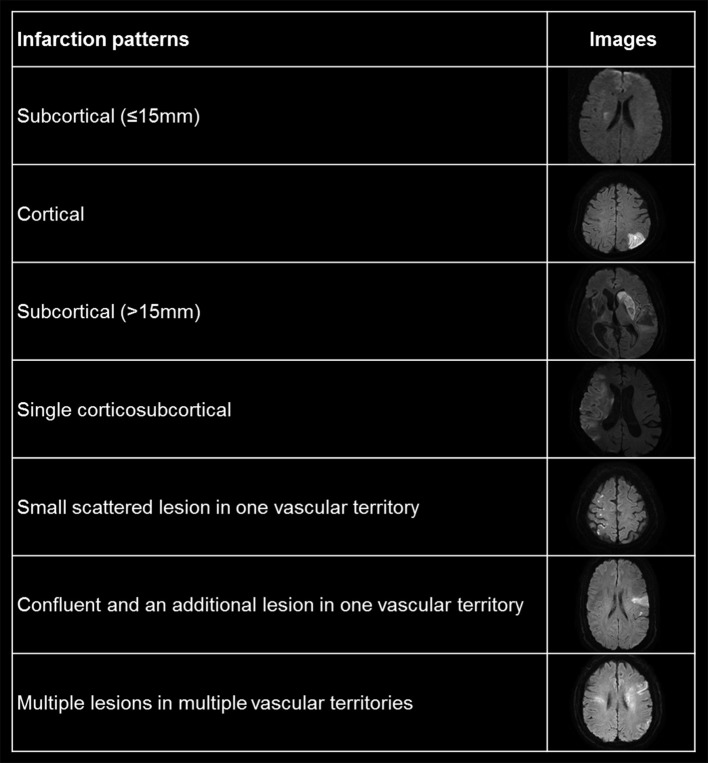
Representative figures for pattern of cerebral infarction in diffusion weighted images.

The following demographics, including vascular risk factors, previous medical history including stroke, underlying ≥50% intra- and/or extracranial atherosclerosis (ICAS, ECAS, respectively), whether recanalization therapy was performed, discharge medication (anti-thrombotics including anti-platelet, warfarin, or non-vitamin K dependent oral anti-coagulant), and modality of recanalization therapy were recorded (11, 12). Stroke severity was determined using the National Institutes of Health Stroke Scale (NIHSS) at admission (13). Stroke classification was determined based on the Trial of Org 10172 in the Acute Stroke Treatment (TOAST) classification system (14). For convenience, stroke subtypes were divided into AF only, AF + large artery atherosclerosis, AF + small vessel occlusion, and AF + stroke of other determined causes. The patterns of cerebral infarction were categorized into subcortical (≤15 mm), cortical, subcortical (>15 mm), single corticosubcortical, small scattered lesion in one vascular territory, confluent, and an additional lesion in one vascular territory and multiple lesions in multiple vascular territories (Figure 1). Presence and classification of hemorrhagic transformation, including symptomatic intracranial hemorrhage, was defined as based on the European Cooperative Acute Stroke Study criteria (15). The image findings of all included patients were validated by an expert group (WKS and BJK). Functional outcome was dichotomized to favorable outcome (mRS ≤ 2) or poor outcome (mRS > 2) at 3 months after index stroke (16). If the patient was found to have died within 3 months, the mRS at 3 months was defined as 6 points. The mRS was routinely checked by a well-trained research nurse or neurology specialist at each hospital.

### Data Management and Quality Control

All data were collected and uploaded via a web-based electronic data capturing system. All investigators accessed this secure data base system and registered mandatory variables related to this study. The collected data were monitored and audited by a quality control team.

### Statistical Analysis

Statistical analysis was performed using the SPSS 22.0 (IBM, Armonk, NY, USA) software program. The data obtained from this clinical study are expressed as the average value, standard deviation or median value and quartile range for continuous data, and frequency and corresponding percentage for categorical data. Independent Student's *t*-test was used for continuous data and the Chi-square test and Fisher's exact test for categorical data. If the parametric test was not satisfactory, we used the appropriate non-parametric test method. Univariate and multivariate binary logistic regression analyses were conducted using the poor outcome at 3 months after index stroke as the dependent variable. To control for over-fitting and small sample size, stroke subtype of AF + stroke for other determined causes was included using the stroke subtype of AF only. Age, sex, and variables with a *p* < 0.05 in univariate analyses and well-known factors related with functional outcome after stroke were entered into multivariate binary and ordinal logistic regression analyses. Because of multicollinearity between underlying risk factors and stratification risk scores, CHADS_2_ score, and CHA_2_DS_2_-VASc score were not entered in the multivariate analysis. A two-tailed *p* < 0.05 was considered significant.

## Results

### Demographics and Characteristics of the Registered Subjects

The baseline characteristics and profiles of vascular risk factors and possible etiologic classification of the subjects are described in Supplementary Table 4. A total of 3,213 stroke patients [mean age, 73.6 ± 9.8 years; men, 50.9% (1,246/3,213)] were included in the study. Etiologic stroke classification among patients who were classified into stroke subtypes was 76.2% (2,450/3,213), the AF only group comprised 83.8% (2,053/2,450), and was followed by two or more causes [atherosclerotic 12.2% (298/2,450), small vessel occlusion 3.0% (74/2,450), and other (1.0% (25/2,450)]. The median National Institute of Health Stroke Scale (NIHSS) score at presentation was 8. The CHADS2 score and CHA2DS2-VASc score were 3.4 ± 0.9 and 4.9 ± 1.3, respectively.

### Factors Associated With Poor Functional Outcome at 3 Months

Among the 3,213 patients included, 959 patients without detailed brain image data [cerebral infarction patterns of diffusion weighed image (*n* = 807), presence of cerebral artery stenosis (*n* = 67), and information for presence of hemorrhagic transformation (*n* = 85)] were excluded. Then, 405 patients who did not have 3-month mRS information were also excluded. A total of 1,849 (57.5%) patients [favorable outcome of 46.9% (868/1,849), poor outcome of 53.1% (981/1,849)] that had prognosis data at 3 months were finally included in our study (Supplementary Figure 2). There were no differences regarding demographics and vascular risk factors among the included patients and non-included patients except frequency of dyslipidemia and persistent AF [more commonly noted in non-included patients; 31.1% vs. 18.6% for dyslipidemia (*p* = 0.001) and 57.4% vs. 45.1% for persistent AF (*p* = 0.001)] (Supplementary Table 5). Compared with non-included (without image data) patients, patients who included in our study had a higher frequency of previous ischemic stroke history (*p* < 0.001). Also, patients who included in our study had a lower frequency of hypercholesterolemia (*p* < 0.001) and stroke subtype of AF only (*p* < 0.001) than those of non-included patients. Considering initial NIHSS and mRS, included patients had higher NIHSS (*p* = 0.013) and mRS (*p* < 0.001) than those with non-included patients (Supplementary Table 6).

Among the 1,849 patients, reperfusion therapies including intravenous t-PA infusion and/or endovascular therapy at index event were performed for 31.5% (584/1,849) of patients. Regarding pre-stroke oral anticoagulant use undergoing reperfusion therapy, frequency of taking warfarin, and NOAC were 12.1%, 4.0% on t-PA, 25.0%, 3.1% for endovascular therapy, and 15.6%, 3.0% on combined therapy (Supplementary Figure 3).

Patients who prescribed antiplatelet at discharge had lower body mass index than that of patients who were not prescribed antiplatelet at discharge (22.7 ± 3.1 vs. 23.2 ± 3.2, *p* = 0.002). Moreover, patients who prescribed antiplatelet at discharge more frequently accompanied under-dosing NOAC at discharge (7.0% vs. 3.7% *p* = 0.008) and ICAS (33.8% vs. 28.3%, *p* = 0.031) than those of patients who were not prescribed antiplatelet at discharge (Supplementary Table 7).

**Figure 2:**
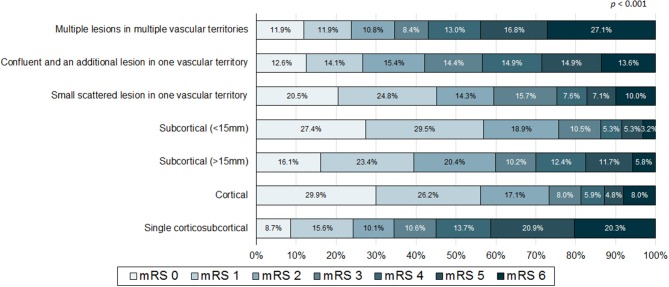
Distribution of modified Rankin score (mRS) on 3 months (percent) according to infarction patterns.

**Figure 3:**
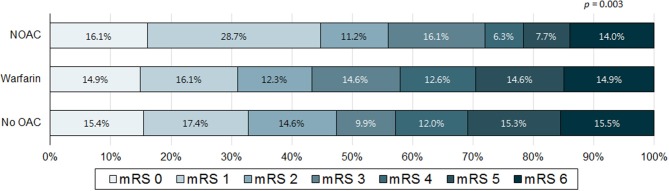
Association of frequency of pre-stroke use of oral anticoagulants (percent) with modified Rankin score (mRS) on 3 months. NOAC, non-vitamin K dependent oral anticoagulant; OAC, oral anticoagulant.

When comparing patients with poor outcome with good outcome, the poor outcome group less frequently included men, had higher NIHSS, pre-stroke mRS, and CHA_2_DS_2_-VASc score, were older and had lower body mass index than that of the good outcome group. Moreover, the poor outcome group more frequently had previous stroke history, congestive heart failure, prior stroke warfarin intake, AF only stroke subtype, asymptomatic ECAS, asymptomatic ICAS, specific infarction patterns (single corticosubcortical, confluent and an additional lesion in one vascular territory, or multiple lesions in multiple vascular territories), presence of hemorrhagic transformation, and thrombolytic therapy. In addition, the poor outcome group less frequently had hypercholesterolemia, current smoking, and prior stroke statin intake (Table 1). Distribution of mRS on 3 months (percent) according to infarction patterns and association of frequency of pre-stroke use of oral anticoagulants (percent) with mRS on 3 months were presented at Figures 2, 3.

**Table 1:** Comparison of clinical and brain image findings according to functional outcome at 3 months after index stroke.

	**mRS ≤ 2 (*n* = 868)**	**mRS > 2 (*n* = 981)**	**Total (*n* = 1,849)**	***p* value**
Demographics				
Sex, male	514 (59.2)	424 (43.2)	938 (50.7)	0.001
Age, years	70.7 ± 9.4	76.1 ± 8.8	73.4 ± 9.6	0.001
Body mass index, kg/m^2^	23.6 ± 3.0	22.8 ± 3.4	23.2 ± 3.2	0.001
Previous stroke				0.001
None	625 (72.0)	619 (63.1)	1,244 (67.3)	
Ischemic stroke	213 (24.5)	306 (31.2)	519 (28.1)	
Hemorrhagic stroke	12 (1.4)	18 (1.8)	30 (1.6)	
Both ischemic and hemorrhagic	4 (0.5)	10 (1.0)	14 (0.8)	
Unknown	14 (1.6)	28 (2.9)	42 (2.3)	
Risk factors				
Congestive heart failure	26 (3.0)	5 (5.4)	79 (4.3)	0.011
Hypertension	595 (68.5)	675 (68.8)	1,270 (68.7)	0.905
Diabetes mellitus	219 (25.2)	265 (27.0)	484 (26.2)	0.384
Hypercholesterolemia	188 (21.7)	156 (15.9)	344 (18.6)	0.002
Coronary artery disease	123 (14.2)	121 (12.3)	244 (13.2)	0.244
Peripheral artery disease	7 (0.8)	13 (1.3)	20 (1.1)	0.369
Current smoking	150 (17.3)	104 (10.6)	254 (13.7)	0.001
Prior medication				
Antiplatelets	367 (42.3)	371 (37.8)	738 (39.9)	0.051
Anticoagulants				0.039
Warfarin	148 (17.1)	194 (19.8)	342 (18.5)	
NOACs	80 (9.2)	63 (6.4)	143 (7.7)	
Statins	193 (22.2)	181 (18.5)	374 (20.2)	0.043
Discharge medication				
Antiplatelets	188 (21.7)	211 (21.5)	399 (21.6)	0.937
Anticoagulants				0.185
Warfarin	586 (67.5)	652 (66.5)	1,238 (67.0)	
NOACs	234 (27.0)	254 (25.9)	488 (26.4)	
Statins	611 (70.4)	683 (69.6)	1,294 (70.0)	0.719
NIHSS	3 [1–8]	14 [9–18]	9 [3–16]	0.001
Pre-stroke mRS	0 [0–1]	0 [0–3]	0 [0–2]	0.001
Type of AF				0.161
Persistent AF	406 (46.8)	427 (43.5)	833 (45.1)	
Paroxysmal AF	462 (53.2)	554 (56.5)	1,016 (54.9)	
Stroke subtype				0.001
AF only	709 (81.7)	839 (85.5)	1,548 (83.7)	
AF + LAA	106 (12.2)	121 (12.3)	227 (12.3)	
AF + SVO	50 (5.8)	11 (1.1)	61 (3.3)	
AF + SOD	3 (0.3)	10 (1.0)	13 (0.7)	
Brain image findings				
Asymptomatic cerebral atherosclerosis				
ECAS	90 (10.4)	226 (23.0)	316 (17.1)	0.001
ICAS	196 (22.6)	349 (35.6)	545 (29.5)	0.001
Infarction pattern on DWI				0.001
Subcortical (≤ 15 mm)	72 (8.3)	23 (2.3)	95 (5.1)	
Cortical	137 (15.8)	47 (4.8)	184 (10.0)	
Subcortical (>15 mm)	82 (9.4)	55 (5.6)	137 (7.4)	
Single corticosubcortical	163 (18.8)	309 (31.5)	472 (25.5)	
Small scattered lesion in one vascular territory	125 (14.4)	85 (8.7)	210 (11.4)	
Confluent and an additional lesion in one vascular territory	161 (18.5)	221 (22.5)	382 (20.7)	
Multiple lesions in multiple vascular territories	128 (14.7)	241 (24.6)	369 (20.0)	
Hemorrhagic transformation				0.001
No hemorrhagic transformation	736 (84.8)	721 (73.5)	1,457 (78.8)	
Hemorrhagic transformation type 1	66 (7.6)	118 (12.0)	184 (10.0)	
Hemorrhagic transformation type 2	35 (4.0)	53 (5.4)	88 (4.8)	
Parenchymal hemorrhage 1	19 (2.2)	48 (4.9)	67 (3.6)	
Parenchymal hemorrhage 2	12 (1.4)	41 (4.2)	53 (2.9)	
Recanalization therapy				0.001
Intravenous	136 (15.7)	185 (18.9)	321 (17.4)	
Intraarterial	40 (4.6)	88 (9.0)	128 (6.9)	
Combined	64 (7.4)	71 (7.2)	135 (7.3)	
CHADS2 score	3 [3–4]	4 [3–4]	3 [3–4]	<0.001
CHA2DS2-VASc score	5 [4–6]	5 [4–6]	5 [4–6]	<0.001

*Data are shown as n (%), mean ± standard deviation, or median [interquartile range]. NOACs, non-vitamin K dependent oral anticoagulants; NIHSS, National Institutes of Health Stroke Scale; mRS, modified Rankin scale; AF, atrial fibrillation; LAA, large artery atherosclerosis; SVO, small vessel occlusion; SOD, stroke of other determined causes; ECAS, extracranial cerebral atherosclerosis; ICAS, intracranial cerebral atherosclerosis; DWI, diffusion weighted image*.

### Independent Factors for Poor Functional Outcome at 3 Months

Multivariate analysis showed increased age [odds ratio (OR): 1.04, 95% confidence interval (CI): 1.03–1.06, *p* = 0.001], previous ischemic stroke history (OR: 1.72, 95% CI: 1.28–2.31, *p* = 0.001), NIHSS (OR: 1.23, 95% CI: 1.20–1.26, *p* = 0.001), pre-stroke mRS (OR: 1.46, 95% CI: 1.32–1.61, *p* = 0.001), presence of asymptomatic ECAS (OR: 1.96, 95% CI: 1.36–2.82, *p* = 0.001), parenchymal hemorrhage type 1 (OR: 2.82, 95% CI 1.43–5.50, *p* = 0.003), and parenchymal hemorrhage type 2 (OR: 3.51, 95% CI: 1.57–7.84, *p* = 0.002) were significantly associated with poor functional outcomes (Table 2). Prior stroke statin intake (OR: 0.69, 95% CI: 0.49–0.98, *p* = 0.038), cortex only located infarction (OR: 0.47, 95% CI: 0.29–0.77, *p* = 0.003), and thrombolytic therapy (intravenous (OR: 0.57, 95% CI: 0.40–0.82) or both (intravenous and intraarterial) (OR: 0.28, 95% CI: 0.17–0.46) were also negatively related to poor functional outcome (Table 2). These associations were consistently noted in ordinal logistic regression analysis including presence of asymptomatic ECAS (OR: 2.04, 95% CI: 1.61–2.58, *p* = 0.001), except prior stroke statin intake (OR: 0.94, 95% CI: 0.74–1.18, *p* = 0.606). Furthermore, in subgroup analysis, there were no statistical interaction between pre-stroke mRS and presence of asymptomatic ECAS (*p* = 0.205), prior stroke statin intake (*p* = 0.580) for poor functional outcome.

**Table 2:** Independent factors for poor functional outcome.

	**Odds ratio (95% confidence interval)**	***p* value**
Demographics		
Sex, male	0.79 (0.59–1.07)	0.137
Age, years	1.04 (1.03–1.06)	0.001
Body mass index, kg/m^2^	0.98 (0.94–1.02)	0.424
Previous stroke		
None	Reference	
Ischemic stroke	1.72 (1.28–2.31)	0.001
Hemorrhagic stroke	1.66 (0.60–4.61)	0.326
Both ischemic and hemorrhagic	2.44 (0.51–11.56)	0.261
Unknown	1.31 (0.52–3.27)	0.555
Risk factors		
Congestive heart failure	1.19 (0.61–2.33)	0.601
Hypercholesterolemia	0.83 (0.58–1.20)	0.725
Smoking	0.97 (0.71–1.34)	0.892
Prior medication		
Anticoagulants		
No anticoagulants	Reference	
Warfarin	1.14 (0.82–1.60)	0.419
NOACs	0.95 (0.59–1.55)	0.862
Statins	0.69 (0.49–0.98)	0.038
Antiplatelet at discharge	1.18 (0. 82–1.62)	0.328
NIHSS	1.23 (1.20–1.26)	0.001
Pre-stroke mRS	1.46 (1.32–1.61)	0.001
Stroke subtype		
AF only	Reference	
AF + LAA	0.90 (0.60–1.34)	0.618
AF + SVO	0.78 (0.32–1.94)	0.604
Brain image findings		
Asymptomatic cerebral atherosclerosis		
ECAS	1.96 (1.36–2.82)	0.001
ICAS	0.91 (0.67–1.21)	0.526
Infarction pattern on DWI		
Subcortical (≤ 15 mm)	Reference	
Cortical	0.47 (0.29–0.77)	0.003
Subcortical (>15 mm)	0.70 (0.41–1.18)	0.186
Single corticosubcortical	0.70 (0.34–1.47)	0.355
Small scattered lesion in one vascular territory	0.73 (0.46–1.14)	0.174
Confluent and an additional lesion in one vascular territory	0.84 (0.57–1.23)	0.382
Multiple lesions in multiple vascular territories	1.23 (0.84–1.81)	0.280
Hemorrhagic transformation		
No hemorrhagic transformation	Reference	
Hemorrhagic transformation type 1	1.52 (0.99–2.33)	0.051
Hemorrhagic transformation type 2	1.31 (0.73–2.35)	0.357
Parenchymal hemorrhage type 1	2.82 (1.43–5.50)	0.003
Parenchymal hemorrhage type 2	3.51 (1.57–7.84)	0.002
Recanalization therapy		
None	Reference	
Intravenous	0.57 (0.40–0.82)	0.003
Intraarterial	0.60 (0.34–1.05)	0.075
Combined	0.28 (0.17–0.46)	0.001

*NOACs, non-vitamin K dependent oral anticoagulants; NIHSS, National Institutes of Health Stroke Scale; mRS, modified Rankin scale; AF, atrial fibrillation; LAA, large artery atherosclerosis; SVO, small vessel occlusion; ECAS, extracranial cerebral atherosclerosis; ICAS, intracranial cerebral atherosclerosis; DWI, diffusion weighted image*.

In subgroup analysis for patients with who were taking anti-thrombotics (antiplatelet, warfarin, and NOAC), multivariate analysis showed that previous ischemic stroke history (OR: 1.80, 95% CI: 1.16–2.80, *p* = 0.008), NIHSS (OR: 1.15, 95% CI: 1.10–1.20, *p* < 0.001), pre-stroke mRS (OR: 1.59, 95% CI: 1.38–1.84, *p* < 0.001), and presence of asymptomatic ICAS (OR: 1.75, 95% CI: 1.16–2.64, *p* = 0.007) were significantly associated with poor functional outcomes (Table 3). The cortex only located infarction (OR: 0.45, 95% CI: 0.20–0.98, *p* = 0.046) and combined intravenous and intraarterial reperfusion therapy (OR: 0.26, 95% CI: 0.11–0.58, *p* = 0.001) were also negatively related to poor functional outcome (Table 3).

**Table 3:** Independent factors for poor functional outcome in patients who taking prior anti-thrombotics.

	**Odds ratio (95% confidence interval)**	***p* value**
Demographics		
Sex, male	0.65 (0.41–1.04)	0.076
Age, years	1.02 (1.00–1.05)	0.016
Body mass index, kg/m^2^	0.95 (0.89–1.02)	0.209
Previous stroke		
None	Reference	
Ischemic stroke	1.80 (1.16–2.80)	0.008
Hemorrhagic stroke	1.73 (0.32–9.37)	0.521
Both ischemic and hemorrhagic	6.49 (0.76–55.50)	0.087
Unknown	1.01 (0.35–2.89)	0.981
Risk factors		
Congestive heart failure	1.88 (0.61–5.82)	0.269
Hypertension	1.10 (0.69–1.76)	0.674
Hypercholesterolemia	0.75 (0.42–1.33)	0.327
Smoking	1.15 (0.70–1.90)	0.568
Prior medication		
Statins	0.91 (0.56–1.48)	0.718
NIHSS	1.15 (1.10–1.20)	<0.001
Pre-stroke mRS	1.59 (1.38–1.84)	<0.001
Stroke subtype		
AF only	Reference	
AF + LAA	1.16 (0.33–4.03)	0.813
AF + SVO	0.58 (0.32–1.07)	0.085
Brain image findings		
Asymptomatic cerebral atherosclerosis		
ECAS	1.53 (0.97–2.42)	0.064
ICAS	1.75 (1.16–2.64)	0.007
Infarction pattern on DWI		
Subcortical (≤ 15 mm)	Reference	
Cortical	0.45 (0.20–0.98)	0.046
Subcortical (>15 mm)	0.75 (0.30–1.71)	0.496
Single corticosubcortical	0.86 (0.25–2.98)	0.817
Small scattered lesion in one vascular territory	0.67 (0.34–1.32)	0.253
Confluent and an additional lesion in one vascular territory	1.16 (0.59–2.26)	0.654
Multiple lesions in multiple vascular territories	1.01 (0.57–1.78)	0.958
Hemorrhagic transformation		
No hemorrhagic transformation	Reference	
Hemorrhagic transformation type 1	1.59 (0.87–2.89)	0.128
Hemorrhagic transformation type 2	1.32 (0.56–3.11)	0.523
Parenchymal hemorrhage type 1	2.12 (0.75–5.95)	0.153
Parenchymal hemorrhage type 2	2.53 (0.80–7.92)	0.111
Recanalization therapy		
None	Reference	
Intravenous	0.60 (0.34–1.07)	0.086
Intraarterial	1.01 (0.38–2.64)	0.979
Combined	0.26 (0.11–0.58)	0.001

*NOACs, non-vitamin K dependent oral anticoagulants; NIHSS, National Institutes of Health Stroke Scale; mRS, modified Rankin scale; AF, atrial fibrillation; LAA, large artery atherosclerosis; SVO, small vessel occlusion; ECAS, extracranial cerebral atherosclerosis; ICAS, intracranial cerebral atherosclerosis; DWI, diffusion weighted image*.

## Discussion

The key findings of our study include the following: (1) to provide demographics and baseline characteristics for a Korean nationwide AF-stroke retrospective study (K-ATTENTION study) and (2) to investigate the independent factors for poor functional outcome at 3 months in this study sample.

Because stroke with AF is independent and different from stroke without AF, stroke subjects with AF should be treated prudently. There are cohort/registries of stroke subjects with AF in some countries other than Korea; however, several limitations exist in these cohort/registries such as small sample size (17), limited inclusion criteria (subjects who received intravenous thrombolytic agents) (18), were prescribed specific NOAC only (19), or warfarin only (20, 21) and lack of information regarding time in therapeutic range in warfarin users (22). In contrast, our K-ATTENTION study has the following significance and strength. First, our study was designed to supply important and timely insight into the management of acute cerebral infarction subjects with AF using a large multicenter, nationwide study. Second, apart from providing information for anticoagulant use and predictive factors for functional outcome, our study provides information on infarction pattern, recanalization therapy outcomes, and detailed anti-thrombotic therapy (particularly NOACs) information for Asian subjects. Third, anticoagulant therapy has important implications for acute stroke patients, including reperfusion therapy. The current guidelines recommend that intravenous tissue plasminogen activator inhibitors should not be administered as often as possible, especially if warfarin concentration is above the certain level or if NOACs are being administered. This means that intra-arterial thrombectomy should be considered immediately in situations requiring recanalization therapy (23, 24). Because our study mainly includes subjects who have been taking anticoagulants, the K-ATTENTION study may provide a current status of mechanical thrombectomy in stroke patients with AF, especially for patients on NOACs. K-ATTENTION study will provide researches with additional information on this part in subsequent studies.

In addition to the well-known factors associated with stroke outcome (age, previous stroke, NIHSS, pre-stroke mRS, infarction pattern, hemorrhagic transformation, and recanalization therapy), prior stroke statin intake was significantly associated with good functional outcomes in our study. A previous study showed that pre-stroke treatment with statins was an independent factor associated with good outcomes in patients with atherosclerotic stroke or lacunar stroke, but not with cardioembolic stroke (25). Additionally, the previous Fukuoka stroke registry-based study, which included cardioembolic stroke, revealed that prior stroke statin treatment in ischemic stroke patients was significantly related to mild neurological symptoms within 24 h of onset (26). Furthermore, a previous meta-analysis including 23,577 patients with AF showed that statin itself was beneficial for incidence or recurrence of AF (27). However, prior stroke statin treatment did not significantly influence the short-term functional outcome (26). In contrast, premorbid use of statins in AF patients is associated with excellent collateral flow in acute stroke patients (28). Our results are partially in line with these studies and any discrepancy may be caused by sample size differences, the inclusion of different patient characteristics or racial differences. Furthermore, we could not analyze the type and intensity of statin, and persistence of statin after admission; therefore, further study is needed.

Our study demonstrated that stenosis or occlusion of the non-relevant artery, especially asymptomatic extra-cranial atherosclerosis, was associated with poor functional outcomes. This may be due to a lack of collateral flow or a decrease in cerebral perfusion, or a recurrent stroke in the non-relevant artery itself. It has been reported that the burden of cerebral atherosclerosis increases with increasing CHADS_2_ score in stroke patients with AF (29). Moreover, another study demonstrated that the presence and increased burden of non-relevant artery stenosis were related to poor functional outcome in acute stroke patients (30). Thus, our data provides additional information that functional outcome may be poor if non-relevant artery stenosis is noted in stroke patients with AF.

In a previous study, severity of stroke at admission and functional outcome of cardioembolic stroke patients managed with NOACs and warfarin within therapeutic ranges were similar, and patient outcomes when taking NOACs were more favorable than those with warfarin below the therapeutic range or those without anticoagulants (31). Furthermore, a recent retrospective observational study of 94,474 patients with AF-stroke demonstrated that taking NOACs before admission was associated with lower odds of moderate to severe stroke and in-hospital mortality (32). Even though significance was only found in univariate analysis; our results demonstrated that taking NOACs before an index stroke might be more beneficial than taking warfarin for short-term functional outcomes. This may be due to the small sample size of subjects taking NOACs prior to an index stroke, or other clinical factors may have had stronger effects on poor functional outcome. Expecting that the prescription of NOACs would increase based on the national insurance coverage system in Korea, further studies regarding this issue are needed.

Our study has limitations. First, because it was performed retrospectively, even though subjects who were included in our study were prospectively collected from a stroke registry from each participating hospital, selection bias exists. Second, because K-ATTENTION was conducted only for subjects who visited regional or local emergency medical centers, it does not reflect the current overall managing status of subjects with AF in Korea. Third, in our dataset, the rate of patients who were lost to follow-up at 3 months is ~18%. This censoring could make selection bias in our study. Fourth, since our study period was not the time when antidote of NOAC was widely used, our study does not provide data on this. Fifth, as with other observational registries, any relationship between treatment and outcomes from K-ATTENTION are based on non-randomized comparisons and thus are potentially influenced by measured and unmeasured confounding factors.

## Conclusions

The K-ATTENTION study provides a valuable opportunity for identifying the recent status of antithrombotic therapy for the secondary prevention of patients with AF-related stroke in Korea. Furthermore, our study suggests that prior stroke statin therapy may be feasible, and the presence of asymptomatic extra-cranial atherosclerosis may affect poor functional outcomes in stroke patient with AF.

## Data Availability Statement

The datasets for this manuscript are not publicly available because: policy of ethical committee of each participating site. Request to access the datasets should be directed to W-KS (mcastenosis@gmail.com).

## Author Contributions

W-KS and T-JS drafted the paper with input and critical review from all authors. I-YB, HW, Y-JK, YC, BK, SH, J-MJ, KO, CK, SY, KP, J-MK, J-HP, JC, M-SP, J-TK, K-HC, Y-HH, J-WC, OB, and G-MK performed study selection, data extraction, and quality assessment.

### Conflict of Interest

W-KS received honoraria for lectures from Pfizer, Sanofi-Aventis, Otsuka Korea, Dong-A Pharmaceutical Co., Ltd., Beyer, Daewoong pharmaceutical Co., Ltd., Daiichi Sankyo Korea Co., Ltd., Boryung pharmaceutical, study grant from Daiichi Sankyo Korea Co., Ltd., consulting fee from OBELAB Inc., and stock option from JLK Inspection. J-MJ received honoraria for lectures from Pfizer, Sanofi-Aventis, Dong-A, Daewoong Pharmaceutical Co., Ltd., and Boryung Pharmaceutical Co., Ltd., a study grant from Il-dong Pharmaceutical Co., Ltd., and consulting fees from OBELAB Inc. The remaining authors declare that the research was conducted in the absence of any commercial or financial relationships that could be construed as a potential conflict of interest.
