# Supplementary material for: Characteristics and Factors for Short-Term Functional Outcome in Stroke Patients With Atrial Fibrillation, Nationwide Retrospective Cohort Study
Source: Front Neurol. 2019 Oct 18;10:1101. doi: 10.3389/fneur.2019.01101 (PMC6813464; doi:10.3389/fneur.2019.01101)
Supplement: Supplementary file 1 [file Data_Sheet_1.doc]

**SUPPLEMENTAL MATERIAL**

**Supplementary Table 1.** Inclusion and exclusion criteria of K-ATTENTION registry.

| Inclusion criteria |
| --- |
| Adults over 20 years of age at screening during study period (from January 1, 2013 to December 31, 2015) |
| During study period, patients who confirmed as cerebral infarction^*^ and accompanying atrial fibrillation^†^ diagnosed via electrocardiogram (ECG), 24 or 48-hour Holter electrocardiogram, or automated rhythm detection device or past or having history of atrial fibrillation |
| Exclusion criteria |
| Subjects who are not adequately screened for stroke and arrhythmia (for example, subjects not performed brain CT and/or MRI, MRA or ECG) and without evidence of cerebral infarction on brain images |

K-ATTENTION: Korean nationwide ATrial fibrillaTion EvaluatioN regisTry in Ischemic strOke patieNts

*: defined as ischemic lesions were identified through brain imaging (CT or MRI) accompanied by neurologic symptoms or signs.

†: defined as arrhythmia that lasts for more than 30 seconds, with irregular R-R intervals and no clear repetitive P-waves

**Supplementary Table 2.** Registries including stroke patients with atrial fibrillation.

|  | **Country** | **Anti-thrombotic agents** | **Setting** | **Patient characteristics** | **Number of included patients** |
| --- | --- | --- | --- | --- | --- |
| CAPTURE | USA | VKA | Multicenter | Stroke/TIA | 1953 |
| FibStroke | Finland | VKA | Multicenter, registry-based | Ischemic stroke/TIA/ICH | 3632 |
| AVAIL | USA | VKA *vs*. antiplatelet | Multicenter, registry-based | Ischemic stroke/TIA | 261 |
| SAMURAI-NVAF | Japan | VKA *vs.* NOACs | Multicenter, registry-based | Acute ischemic stroke/TIA | 1192 |
| daVinci | Japan | VKA *vs.* NOACs | Multi-center, prospective | Acute ischemic stroke/TIA & IV-tPA | 235 |
| RELAXED | Japan | Rivaroxaban only | Multicenter, registry-based | Acute phase of NVAF-related stroke | 2000 |
| K-ATTENTION  (our study) | Korea | VKA *vs.* NOACs *vs.* antiplatelet | Multicenter, registry-based | Acute ischemic stroke/TIA | 3213 |

VKA: vitamin K antagonist, TIA: transient ischemic attack, ICH: intracerebral hemorrhage, NOACs: non–vitamin K antagonist oral anticoagulants, IV-tPA: intravenous- tissue plasminogen activator

CAPTURE: Am J Prev Med. 2006 Dec;31(6 Suppl 2):S224-9, FibStroke: Int J Cardiol. 2017 Jan 15;227:869-874, AVAIL: Stroke. 2011 Dec;42(12):3477-83, SAMURAI-NVAF: Int J Stroke. 2015 Aug;10(6):836-42, daVinci: J Thromb Thrombolysis. 2016 Nov;42(4):453-62, RELAXED: J Stroke Cerebrovasc Dis. 2016 Jun;25(6):1342-8.

**Supplementary Table 3.** Primary, secondary, and exploratory purpose of K-ATTENTION study.

| Primary |
| --- |
| Investigation of current status and trends of antithrombotic therapy for secondary prevention of in stroke patients with atrial fibrillation |
| Comparison of incidence of major cardiovascular events, including cerebral infarction, and systemic embolism for secondary prevention in stroke patients with atrial fibrillation |
| Development of predictive model of major cardiovascular events, including cerebral infarction, and systemic embolism in stroke patients with atrial fibrillation |
| Secondary |
| Characteristics of cerebral infarction including severity, hemorrhagic transformation, and recanalization therapy according to prior medication including antithrombotics in stroke patients with atrial fibrillation |
| Reperfusion rate, grade (thrombolysis in cerebral infarction), efficacy, safety of recanalization therapy according to prior medication including antithrombotics in stroke patients with atrial fibrillation |
| Comparison of recurrence / aggravation of cerebral infarction according to acute phase antithrombotic therapy in stroke patients with atrial fibrillation |
| Investigation of current status and trends of antithrombotic therapy according to comorbidity (coronary artery diseases, chronic kidney diseases, liver disease, malignancy, and asymptomatic cerebral atherosclerosis) |
| Occurrence of major cardiovascular events / cerebral hemorrhage / major bleeding incident according to time in therapeutic range of warfarin in stroke patients with atrial fibrillation |
| Characteristics of infarction pattern, acute treatment methods, effectiveness and safety of antithrombotics in recurrent stroke in stroke patients with atrial fibrillation |
| Occurrence of major cardiovascular according to medication discontinuation or switching in stroke patients with atrial fibrillation |
| Exploratory |
| Association of major cardiovascular events with biomarkers (free fatty acid, B-type natiuretic peptide, D-dimer, fibrinogen, etc.) in stroke patients with atrial fibrillation |
| Association of major cardiovascular events with parameters of electrocardiogram, 24 or 48-hour Holter electrocardiogram, or automated rhythm detection device in stroke patients with atrial fibrillation |

**Supplementary Table 4.** Demographics, clinical variables and brain image findings of total patients who included K-ATTENTION registry

| **Variables** | **Data Description** |
| --- | --- |
| Demographics |  |
| Sex, male | 50.9% (1246/3213) |
| Age, years | 73.6 ± 9.6 (n = 3213) |
| Body mass index, kg/m^2^ | 23.3 ± 3.4 (n = 3213) |
| Previous stroke data |  |
| None | 68.5% (2202/3213) |
| Ischemic stroke | 27.2% (875/3213) |
| Hemorrhagic stroke | 1.7% (55/3213) |
| Both ischemic and hemorrhagic | 0.8% (25/3213) |
| Unknown | 1.7% (56/3213) |
| Risk factors data |  |
| Congestive heart failure | 3.3% (107/3213) |
| Hypertension | 53.0% (1702/3213) |
| Diabetes mellitus | 20.4% (657/3213) |
| Hypercholesterolemia | 17.1% (550/3213) |
| Coronary artery disease | 9.6% (307/3213) |
| Peripheral artery disease | 0.8% (25/3213) |
| Current smoking | 10.4% (335/3213) |
| Prior medication data |  |
| Antiplatelets | 41.9% (1349/3213) |
| Anticoagulants |  |
| Warfarin | 19.5% (442/2263) |
| NOACs | 7.6% (173/2263) |
| Statins | 21.9% (635/2890) |
| Discharge medication data |  |
| Antiplatelets | 21.7% (630/2890) |
| Anticoagulants |  |
| Warfarin | 70.0% (2023/2890) |
| NOACs | 26.9% (780/2890) |
| Statins | 73.0% (2110/2890) |
| Initial NIHSS | 8 [2 – 15] (n = 3191) |
| Initial mRS | 3 [1 – 5] (n = 2302) |
| Type of AF | 99.9% (3210/3213) |
| Persistent AF | 50.3% (1614/3210) |
| Paroxysmal AF | 49.7% (1596/3210) |
| Stroke subtype data | 76.2% (2450/3213) |
| AF only | 83.8% (2053/2450) |
| AF + LAA | 12.2% (298/2450) |
| AF + SVO | 3.0% (74/2450) |
| AF + SOD | 1.0% (25/2450) |
| Brain image findings data |  |
| Asymptomatic cerebral atherosclerosis |  |
| ECAS | 17.1% (457/2672) |
| ICAS | 29.3% (795/2709) |
| Infarction pattern on DWI data | 74.7% (2401/3213) |
| Subcortical (≤ 15mm) | 4.1% (98/2401) |
| Cortical | 8.0% (192/2401) |
| Subcortical (> 15mm) | 6.0% (144/2401) |
| Single corticosubcortical | 20.2% (486/2401) |
| Small scattered lesion in one vascular territory | 9.2% (222/2401) |
| Confluent and an additional lesion in one vascular territory | 17.1% (410/2401) |
| Multiple lesions in multiple vascular territory | 16.2% (390/2401) |
| Hemorrhagic transformation data | 65.4% (2102/3213) |
| No hemorrhagic transformation | 80.6% (1695/2102) |
| Hemorrhagic transformation type 1 | 9.0% (190/2102) |
| Hemorrhagic transformation type 2 | 4.2% (89/2102) |
| Parenchymal hemorrhage 1 | 3.3% (70/2102) |
| Parenchymal hemorrhage 2 | 2.8% (58/2102) |
| Recanalization therapy data | 76.2% (2450/3213) |
| Intravenous | 17.2% (422/2450) |
| Intraarterial | 6.4% (156/2450) |
| Both | 6.7% (165/2450) |
| CHADS_2_ score | 3.4 ± 0.9 (n = 3213) |
| CHA_2_DS_2_-VASc score | 4.9 ± 1.3 (n = 3213) |

Data are presented as % (number of cases/number of informed cases) or mean ± standard deviation (number of informed cases) or median [interquartile range] (number of informed cases).

NOACs: non-vitamin K dependent oral anticoagulants, NIHSS: National Institutes of Health Stroke Scale, mRS: modified Rankin scale, AF: atrial fibrillation, LAA: large artery atherosclerosis, SVO: small vessel occlusion, SOD: stroke of other determined causes, ECAS: extracranial cerebral atherosclerosis, ICAS: intracranial cerebral atherosclerosis, DWI: diffusion weighted image.

**Supplementary Table 5. Comparison of demographics and risk factors between included patients and non-included patients.**

|  | **Included**  **(n = 1849)** | **Non included**  **(n = 1364)** | ***p* value** |
| --- | --- | --- | --- |
| Demographics |  |  |  |
| Sex, male | 938 (50.7) | 712 (52.2) | 0.410 |
| Age, years | 73.4 ± 9.6 | 73.6 ± 10.2 | 0.299 |
| Body mass index, kg/m^2^ | 23.2 ± 3.2 | 23.3 ± 3.3 | 0.478 |
| Previous stroke |  |  | 0.053 |
| None | 1244 (67.3) | 958 (70.2) |  |
| Ischemic stroke | 519 (28.1) | 356 (26.1) |  |
| Hemorrhagic stroke | 30 (1.6) | 25 (1.8) |  |
| Both ischemic and hemorrhagic | 14 (0.8) | 11 (0.8) |  |
| Unknown | 42 (2.3) | 14 (1.0) |  |
| Risk factors |  |  |  |
| Congestive heart failure | 79 (4.3) | 62 (4.5) | 0.709 |
| Hypertension | 1270 (68.7) | 957 (70.2) | 0.370 |
| Diabetes mellitus | 484 (26.2) | 372 (27.3) | 0.487 |
| Hypercholesterolemia | 344 (18.6) | 424 (31.1) | 0.001 |
| Coronary artery disease | 244 (13.2) | 171 (12.5) | 0.582 |
| Peripheral artery disease | 20 (1.1) | 18 (1.3) | 0.537 |
| Current smoking | 254 (13.7) | 176 (12.9) | 0.493 |
| Persistent AF | 833 (45.1) | 781 (57.4) | 0.001 |
| CHA₂DS₂-VASc score | 5 [4 – 6] | 5 [4 – 6] | 0.484 |

Data are presented as number (%) or mean ± standard deviation. AF: atrial fibrillation,

**Supplementary Table 6. Comparison of demographics and risk factors between included patients and non-included patients.**

|  | **Included**  **(n = 1849)** | **Non included**  **(No image data)**  **(n = 959)** | **Non included**  **(No 3 months mRS data)**  **(n = 405)** | ***p* value** |
| --- | --- | --- | --- | --- |
| Demographics |  |  |  |  |
| Sex, male | 938 (50.7) | 489 (51.0) | 217 (53.6) | 0.578 |
| Age, years | 73.4 ± 9.6 | 73.8 ± 9.8 | 73.8 ± 10.7 | 0.479 |
| Body mass index, kg/m^2^ | 23.2 ± 3.2 | 23.5 ± 3.6 | 23.1 ± 3.3 | 0.118 |
| Previous stroke |  |  |  | <0.001 |
| None | 1244 (67.3) | 704 (73.4) | 255 (63.0) |  |
| Ischemic stroke | 519 (28.1) | 217 (22.6) | 138 (34.1) |  |
| Hemorrhagic stroke | 30 (1.6) | 22 (2.3) | 3 (0.7) |  |
| Both ischemic and hemorrhagic | 14 (0.8) | 7 (0.7) | 4 (1.0) |  |
| Unknown | 42 (2.3) | 9 (0.9) | 5 (1.2) |  |
| Risk factors |  |  |  |  |
| Congestive heart failure | 79 (4.3) | 44 (4.6) | 19 (4.7) | 0.891 |
| Hypertension | 1270 (68.7) | 682 (71.1) | 269 (66.4) | 0.189 |
| Diabetes mellitus | 484 (26.2) | 284 (29.6) | 95 (23.5) | 0.038 |
| Hypercholesterolemia | 344 (18.6) | 306 (31.9) | 118 (29.1) | <0.001 |
| Coronary artery disease | 244 (13.2) | 104 (10.8) | 63 (15.6) | 0.043 |
| Peripheral artery disease | 20 (1.1) | 13 (1.4) | 7 (1.7) | 0.531 |
| Current smoking | 254 (13.7) | 128 (13.3) | 43 (10.6) | 0.242 |
| Initial NIHSS | 9 [3 – 16] | 7 [2 – 15] | 6 [2 – 16] | 0.013 |
| Initial mRS | 4 [2 – 5] | 1 [0 – 3] | 3 [1 – 5] | <0.001 |
| Stroke subtype data |  |  |  | <0.001 |
| AF only | 1548 (83.7) | 815 (85.0) | 352 (86.9) |  |
| AF + LAA | 227 (12.3) | 120 (12.5) | 27 (6.7) |  |
| AF + SVO | 61 (3.3) | 13 (1.4) | 10 (2.5) |  |
| AF + SOD | 13 (0.7) | 11 (1.1) | 16 (4.0) |  |
| CHADS_2_ score | 3 [3 – 4] | 4 [3 – 4] | 3 [3 – 4] | 0.143 |
| CHA_2_DS_2_-VASc score | 5 [4 – 6] | 5 [4 – 6] | 5 [4 – 6] | 0.360 |

Data are presented as number (%) or mean ± standard deviation or median [interquartile range]. NIHSS: National Institutes of Health Stroke Scale, mRS: modified Rankin scale, AF: atrial fibrillation, LAA: large artery atherosclerosis, SVO: small vessel occlusion, SOD: stroke of other determined causes.

Comparison of age and body mass index was performed with One-way ANOVA test with Bonferroni’s post-hoc analysis.

Comparison of initial NIHSS, initial mRS, CHADS_2_ score, and CHA_2_DS_2_-VASc score were performed with Kruskal-Wallis test.

**Supplementary Table 7.** Comparison of clinical and image findings according to antiplatelet prescription at discharge.

|  | **Antiplatelet at discharge (-)**  **(n = 1450)** | **Antiplatelet at discharge (+)**  **(n = 399)** | ***p* value** |
| --- | --- | --- | --- |
| Demographics |  |  |  |
| Sex, male | 739 (51.0) | 199 (49.9) | 0.700 |
| Age, years | 73.4 ± 9.7 | 73.6 ± 9.5 | 0.695 |
| Body mass index, kg/m^2^ | 23.2 ± 3.2 | 22.7 ± 3.1 | 0.002 |
| Previous stroke |  |  | 0.130 |
| None | 955 (65.9) | 289 (72.4) |  |
| Ischemic stroke | 427 (29.4) | 92 (23.1) |  |
| Hemorrhagic stroke | 25 (1.7) | 5 (1.3) |  |
| Both ischemic and hemorrhagic | 11 (0.8) | 3 (0.8) |  |
| Unknown | 32 (2.2) | 10 (2.5) |  |
| Risk factors |  |  |  |
| Congestive heart failure | 64 (4.4) | 15 (3.8) | 0.567 |
| Hypertension | 988 (68.1) | 282 (70.7) | 0.333 |
| Diabetes mellitus | 383 (26.4) | 101 (25.3) | 0.658 |
| Hypercholesterolemia | 278 (19.2) | 66 (16.5) | 0.232 |
| Coronary artery disease | 182 (12.6) | 62 (15.5) | 0.118 |
| Peripheral artery disease | 15 (1.0) | 5 (1.3) | 0.708 |
| Current smoking | 201 (13.9) | 53 (13.3) | 0.485 |
| Under-dosing NOAC at discharge | 54 (3.7) | 28 (7.0) | 0.008 |
| Type of AF |  |  | 0.118 |
| Persistent AF | 667 (46.0) | 166 (41.6) |  |
| Paroxysmal AF | 783 (54.0) | 233 (58.4) |  |
| Stroke subtype |  |  | 0.053 |
| AF only | 1231 (84.9) | 317 (79.4) |  |
| AF + LAA | 163 (11.2) | 64 (16.0) |  |
| AF + SVO | 47 (3.2) | 14 (3.5) |  |
| AF + SOD | 9 (0.6) | 4 (1.0) |  |
| Brain image findings |  |  |  |
| Asymptomatic cerebral atherosclerosis |  |  |  |
| ECAS | 242 (16.7) | 74 (18.5) | 0.383 |
| ICAS | 410 (28.3) | 135 (33.8) | 0.031 |
| Infarction pattern on DWI |  |  | 0.553 |
| Subcortical (≤ 15mm) | 372 (25.7) | 100 (25.1) |  |
| Cortical | 152 (10.5) | 32 (8.0) |  |
| Subcortical (> 15mm) | 101 (7.0) | 36 (9.0) |  |
| Single corticosubcortical | 73 (5.0) | 22 (5.5) |  |
| Small scattered lesion in one vascular territory | 159 (11.0) | 51 (12.8) |  |
| Confluent and an additional lesion in one vascular territory | 301 (20.8) | 81 (20.3) |  |
| Multiple lesions in multiple vascular territory | 292 (20.1) | 77 (19.3) |  |
| Hemorrhagic transformation |  |  | 0.812 |
| No hemorrhagic transformation | 1139 (78.6) | 318 (79.7) |  |
| Hemorrhagic transformation type 1 | 149 (10.3) | 35 (8.8) |  |
| Hemorrhagic transformation type 2 | 70 (4.8) | 18 (4.5) |  |
| Parenchymal hemorrhage 1 | 53 (3.7) | 14 (3.5) |  |
| Parenchymal hemorrhage 2 | 39 (2.7) | 14 (3.5) |  |
| CHADS₂score | 3 [3 – 4] | 4 [3 – 4] | 0.616 |
| CHA₂DS₂-VASc score | 5 [4 – 6] | 5 [4 – 6] | 0.794 |

Data are shown as n (%), mean ± standard deviation, or median [interquartile range].

NOACs: non-vitamin K dependent oral anticoagulants, AF: atrial fibrillation, LAA: large artery atherosclerosis, SVO: small vessel occlusion, SOD: stroke of other determined causes, ECAS: extracranial cerebral atherosclerosis, ICAS: intracranial cerebral atherosclerosis, DWI: diffusion weighted image.

**Supplementary Figure 1**. Distribution of participating centers in the K-ATTENTION registry.


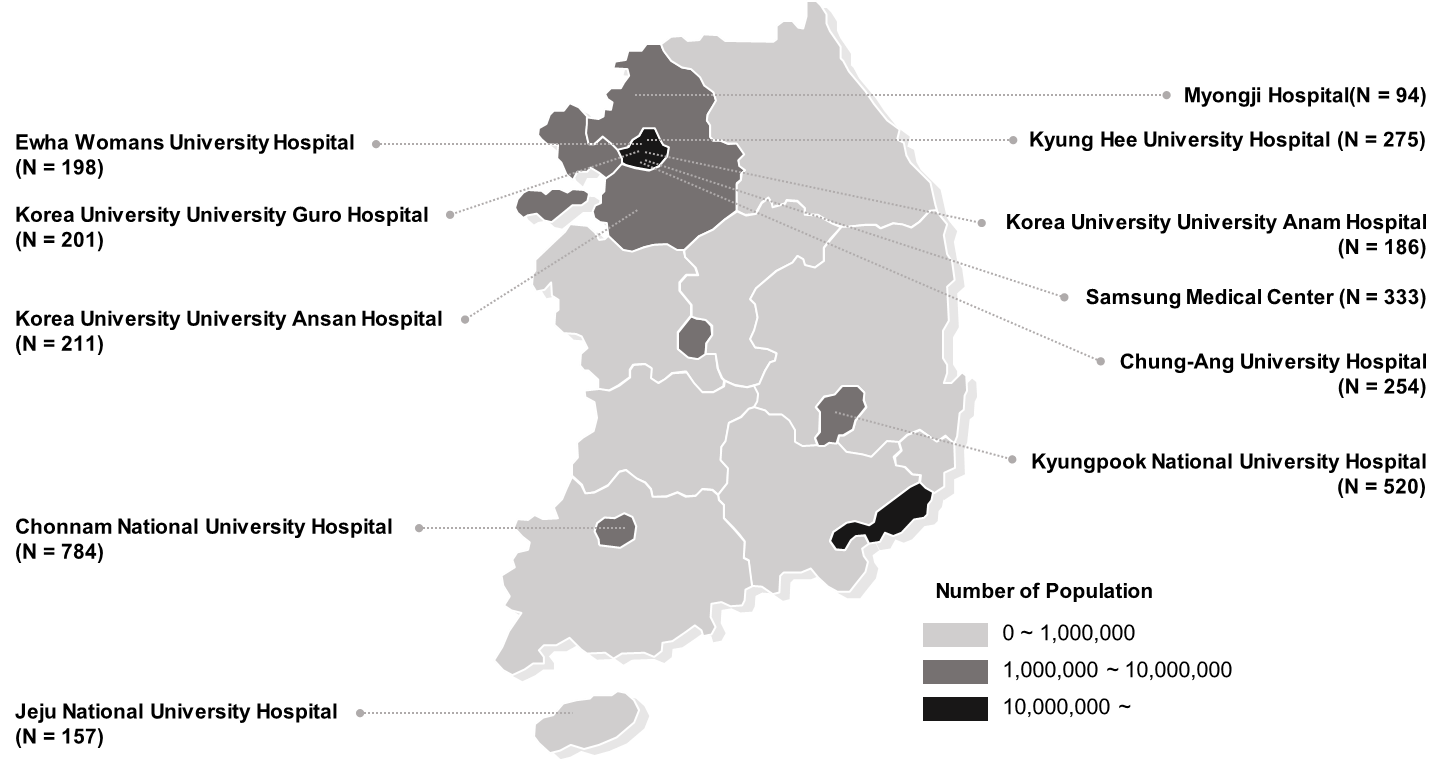


**Supplementary Figure 2.** Flow chart depicting the participation of subjects in our study.





**Supplementary Figure 3.** Proportion of anticoagulants according to receiving recanalization procedures.

**
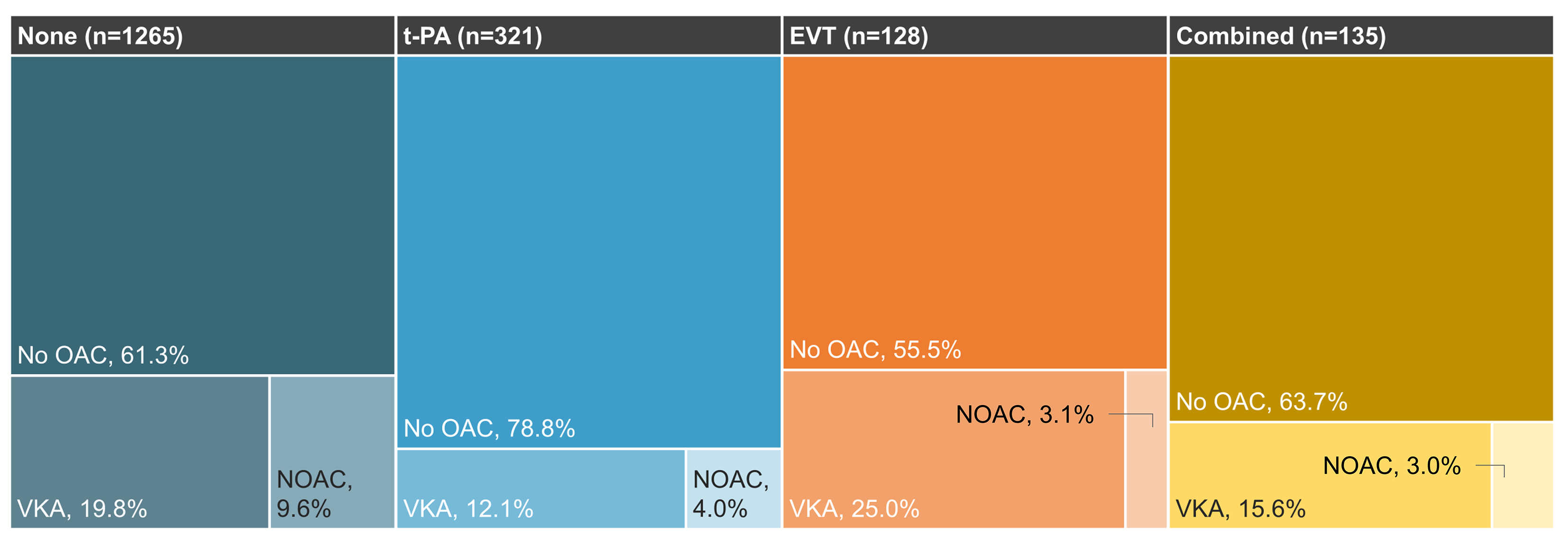
**

The percent and size of box mean proportion of anticoagulants according to receiving recanalization procedures. Combinded means patients who received both intra-venous and intra-arterial thrombolysis. EVT: endovascular treatment, OAC: oral anticoagulant, VKA: vitamin K antagonist, NOAC: non-vitamin K dependent oral anticoagulant.
